# Cognitive behavioral group treatment for low self-esteem in psychosis: a proof of concept study

**DOI:** 10.1186/s12888-021-03579-3

**Published:** 2021-11-12

**Authors:** Elisabeth C. D. van der Stouwe, Chris N. W. Geraets, Mirjam Rutgers, Wim Veling

**Affiliations:** Department of Psychiatry, University of Groningen, University Medical Center Groningen, PO Box 30.001, Groningen, RB 9700 the Netherlands

**Keywords:** Self-esteem, Psychosis, Treatment, Intervention, CBT, Group therapy, Depression

## Abstract

**Background:**

Patients with a psychotic disorder often suffer from low self-esteem, which has been related to higher suicidal risk, poor quality of life and, the maintenance of psychotic and depression symptoms. However, intervention studies are scarce and reported interventions concern individual therapies provided by highly educated psychologists. Both the individual setting and the required qualifications of the therapist may contribute to a low level of availability of an intervention. Therefore we aimed to investigate the efficacy of an easily accessible psychological group treatment targeting self-esteem in patients with a psychotic disorder.

**Methods:**

Thirty patients with a psychotic disorder were included in this pilot study. All participants received nine weekly group sessions of 90 min. The therapy was offered in groups of six to eight patients and was provided by a psychiatry nurse and a graduate psychologist. To assess self-esteem the Rosenberg Self-esteem Scale and the Self-Esteem Rating Scale were used, to measure depression symptoms the Beck Depression Inventory-II was administered. Questionnaires were completed at baseline and post-treatment.

**Results:**

Twenty-seven patients (90%) completed treatment. At post-treatment, self-esteem was significantly increased and depression symptoms were significantly decreased compared to baseline.

**Discussion:**

This pilot study demonstrates the feasibility and treatment potential of a self-esteem group treatment provided by a psychiatry nurse and graduate psychologist in a patient population that receives little psychological treatment. Results suggest that this easily accessible intervention may be effective in improving self-esteem and reducing depression symptoms.

## Introduction

Low self-esteem is highly prevalent among patients with a psychotic spectrum disorder, and may be a consequence of symptoms, loss of social roles, experiences of failure, and (self) stigma [1–3]. In turn, low self-esteem has been implicated in the development and maintenance of psychotic symptoms [4] as well as depressive symptoms [5, 6]. Furthermore, low self-esteem has been related to higher suicidal risk [7], more relapses [8], and poor quality of life [9] in psychosis. Therefore, effective interventions targeted at self-esteem in patients with a psychotic disorder are of utmost importance.

Up until now, only five studies investigated the effect of psychological interventions on self-esteem in patients with a psychotic disorder. While three of these (pilot) studies reported positive effects on self-esteem [6, 10, 11], others found no such effect [12, 13]. The investigated psychological treatments have similarities but differ with regard to their focus. Sönmez et al. (2020) [12] used an intervention that is mainly a cognitive approach including reappraisal of negative self-cognitions. Steel et al. (2015) [13] and van der Gaag et al. (2012) [6] used positive or competitive memory training (COMET [14, 15];) which posits that there are functional self-cognitions available, but these need to become more prominent by imagery of positive memories. Therefore, COMET primarily focuses on emotion. Finally, Freeman et al. (2014) [10] and Hall & Tarrier (2003) [11] focused on behavior, by directing selective attention towards concrete positively evaluated behavior using a positive diary. To summarize, the few investigated treatment protocols show similarities but emphasize different elements, with concrete behavioral elements possibly being most suitable for people with psychosis.

With regard to the context of the interventions, most of the reported treatments were provided by highly educated, specialized (clinical) psychologists or psychologists who received (substantial) training in the specific treatment protocol [10–12]. Moreover, all interventions were individual treatments. Both the level of education of the therapist and the individual setting may contribute to a low level of availability of an intervention for a patient population that already receives little psychological treatment [16]. Despite cognitive-behavioral therapy (CBT) in the treatment guidelines, in the Netherlands approximately 70–75% of patients with a psychotic disorder have no access to psychological treatment.

Group therapy led by therapists with less specialized training may be suitable to fill the gap. Therefore we aimed to investigate the efficacy of an accessible psychological group treatment [17] targeting self-esteem in patients with a psychotic disorder. The treatment has the potential to be easily available because of the group setting, the required level of education of the therapists, and the clear and structured treatment program. In line with previous effective interventions, the current study used a psychological treatment focused on concrete behavioral elements. We hypothesize that this psychological group treatment has a positive effect on self-esteem and depression symptoms.

## Methods

### Participants & procedure

Participants were patients from the Psychosis department of the University Center of Psychiatry (UCP), which is part of the University Medical Center Groningen (UMCG) located in the Netherlands. Patients that are referred to the Psychosis department are invited for one or more intake sessions with a team consisting of at least a psychiatrist. Additionally, patients are asked to complete online questionnaires regarding symptoms and quality of life. Furthermore, patients are formally diagnosed by means of the miniSCAN [18] by a psychologist specialized in this instrument. The miniSCAN is a short version of the Schedule for Clinical Assessement in Neuropsychiatry and consists of structured interview questions regarding axis-I symptoms of the DSM-IV. All included participants were diagnosed with a psychotic spectrum disorder according to DSM-IV or DSM 5. As part of the treatment program at the UCP, patients were referred to the self-esteem group training in case of low self-esteem, indicated by their clinician. Subsequently, an intake was scheduled during which the therapists (psychiatry nurse and/or psychologist) provided the patient with more information about the therapy, inventoried the patient’s negative core beliefs, and assessed the patient’s motivation. Before the start (T0) and after the 9-session group therapy (T1), participants completed three questionnaires to determine the treatment effect. In the current study, data from patients that participated in the group therapy between 2014 and 2020 were used. There was no data available from 2017 and 2018. From all of the participant’s written informed consent was obtained. The current research was not reviewed by a medical ethical board, because this study concerned the evaluation of regular care at the UCP.

### Intervention

The group therapy consisted of 9 weekly sessions of 90 min including a short break. The therapy was offered in groups of six to eight patients and was given by a psychiatry nurse (trained in cognitive behavioral therapy) and a graduate psychologist (a master’s degree). The graduate psychologists were in training to become a post-graduate psychologist. Throughout the study, one psychiatry nurse and five different graduate psychologists conducted the therapy groups. The CBT protocol developed by De Neef (2010) [17] was used. This therapy is based on schema theory, which posits that throughout their development people acquire certain schemas, which are cognitive frameworks based on past experience and personally relevant memories. Schemas help to organize information and allow to take shortcuts in interpreting a vast amount of information. Dysfunctional negative self-schemas induce emotional biases in attention, interpretation, and memory towards negative information that is in line with the negative self-beliefs. The therapy aims to strengthen pathways to positive self-beliefs and make them more easily accessible. Throughout the therapy, the attention and memory are drawn to positive events and a patient’s qualities by repeatedly discussing or noting them in the group sessions and by keeping a diary of positive events and corresponding qualities. The dominant behavioral element of the therapy concerns practicing with new behavior which is in line with positive self-beliefs by dismissing disabling precepts.

The protocol provides a clear and structured program that consists of several subsequent steps. The intervention is designed to provide concrete and simple exercises for patients to integrate into their daily lives. First, patients receive psycho-education, and the costs and benefits of negative and positive self-beliefs are discussed to increase motivation for change. Next, a new positive self-belief is formulated which is antagonistic to the patient’s core negative belief. Throughout the intervention, this positive self-belief is measured to monitor progress. Following, the positive diary is introduced. Positive events are connected to personal qualities that are all gathered on a personal quality list. In line with their qualities and positive self-belief, desired behavior is inventoried and practiced. Finally, patients receive information and training about receiving criticism and they discuss how to prevent relapse.

### Primary outcome measure

#### RSES

The Rosenberg Self-Esteem Scale (RSES [19];) is the most frequently used self-report instrument assessing global self-esteem. The scale consists of 10 items rated on a 4-point Likert scale (0 = “strongly disagree” to 3 = “strongly agree”). Higher scores indicate a higher level of self-esteem (range 0–30). The scale has high internal consistency and good test-retest reliability and construct validity [20]. Although the RSES may be viewed as the gold standard when it comes to the assessment of self-esteem, the instrument may be not so sensitive to change [21].

#### Sers

Self-esteem was also assessed with the Self-Esteem Rating Scale - Short Form (SERS-SF [21];). The self-report scale consists of 20 items rated on a 7-point (1 = “never” to 7 = “always”) scale. The SERS-SF comprises two subscales, positive and negative self-esteem, which were used separately in the analyses. Higher scores on the positive subscale indicate a higher level of self-esteem (range 0–70), higher scores on the negative subscale indicate a lower level of self-esteem (0–70). The scale has good internal consistency, good test-retest reliability, and convergent validity in patients with psychosis [21].

### Secondary outcome measure

#### BDI-ii

Current level of depression was assessed with the Beck Depression Inventory-II (BDI-II [22];. The BDI-II is a widely used self-report inventory that consists of 21 items that are rated on a four-point scale (0–3). Higher scores indicate greater symptom severity (range 0–63). The inventory has high internal consistency and test-retest reliability [23].

### Statistical analysis

The data were visually inspected and assumptions were checked. Two-sided paired samples t-tests were performed for each of the outcome variables on all complete cases (patients who completed both the T0 and T1 assessment) to examine the effect of the group therapy on levels of (positive and negative) self-esteem and depression symptomatology. Analyses were conducted with SPSS version 23.0. A *p*-value below 0.05 was considered statistically significant.

## Results

### Sample characteristics

Characteristics of the sample are presented in Table 1. Thirty patients (fifteen male) with a mean age of 32.5 years (SD = 9.5) participated in the study. Thirteen of the participants were diagnosed with schizophrenia and seventeen of the participants were diagnosed with another type of psychotic spectrum disorder. Three patients dropped out of treatment (two male, mean age 28.3 years old, SD = 3.2).

**Table 1:** Demographic and clinical characteristics

	Participants
n	30
Age mean (SD)	32.5 (9.5)
Gender male n (%)	15 (50.0%)
DSM diagnosis^a^	
Schizophrenia	9 (31.0%)
Schizoaffective disorder	7 (24.1%)
Delusional disorder	1 (3.4%)
Psychotic disorder not otherwise specified	12 (41.4%)
Comorbid diagnosis^a^	
Depressive disorder	5 (17.2%)
Substance use disorder	4 (13.8%)
Borderline personality disorder	2 (6.8%)
Dependent personality disorder	2 (6.8%)
Autism spectrum disorder	1 (3.4%)
None	18 (62.0%)
Medication use^a^	
Antipsychotics	29 (100%)
Aripiprazole	10 (34.5%)
Cisordinole	1 (3.4%)
Clozapine	6 (20.7)
Haloperidole	1 (3.4%)
Olanzapine	6 (20.7%)
Pimozide	1 (3.4%)
Quetiapine	8 (27.5%)
Risperidone	9 (31.0%)
Olanzapine equivalent of prescribed antipsychotic medication (mean, mg per day)	9.9 (5.0)
Antidepressants	8 (27.6%)
Citalopram	2 (6.8%)
Clomipramine	1 (3.4%)
Mirtazepine	1 (3.4%)
Nortryptiline	2 (6.8%)
Venlafaxine	2 (6.8%)
Outpatient ^a^	26 (89.7%)
Completed education ^a^	
Primary education	0 (0%)
Secondary education	10 (34.5%)
Vocational education	8 (27.6%)
Higher	11 (38.0%)
Employment status ^a^	
Paid work	9 (31.0%)
Volunteer work	7 (24.1%)
Unemployed	7 (24.1%)
Study	6 (20.7%)
Relationship status ^a^	
Relationship	6 (20.7%)
Single	23 (79.3%)

^a^For these characteristics, data of 29 instead of 30 patients could be obtained

Data are n (%) or mean (SD).

### Outcome measures

Results are presented in Table 2. On average, self-esteem assessed with the RSES improved with 7.1 points in patients after the therapy. Similarly, the SERS showed an average improvement of 9.6 (6.9–12.3) points on the positive self-esteem subscale and 7.7 (2.1–13.2) points on the negative self-esteem subscale at post-treatment. With regard to depression symptoms, our results suggest an average 7.8 (3.1–11.8) point improvement following treatment.

**Table 2:** Means, standard deviations, and test results of outcomes over time

	Baseline Mean (*SD*)	Post-treatment Mean (SD)	Paired difference (CI)	*t* (df)	*p*
**RSES**	*n = 27*	*n = 26*			
	12.4 (5.0)	19.2 (3.9)	7.1 (4.7–9.4)	6.3 (23)	< 0.001
**SERS**	*n = 26*	*n = 24*			
Subscale positive	37.4 (9.6)	46.7 (9.0)	9.6 (6.9–12.3)	7.5 (22)	< 0.001
Subscale negative	44.5 (11.4)	35.5 (10.2)	−7.7 (−2.1 – −13.2)	− 2.9 (22)	0.009
**BDI-II**	*n = 25*	*n = 25*			
	21.5 (12.6)	13.3 (9.9)	−7.8 (−3.11 – −11.8)	−4.1 (20)	0.001

## Discussion

This was the first study investigating a self-esteem therapy provided by a psychiatry nurse and a graduate psychologist in a group setting in patients with a psychotic disorder. Therapy drop-out rates were low; twenty-seven out of thirty patients (90%) completed the therapy, which indicates that the group therapy was well accepted. After the therapy, patients reported increased self-esteem and decreased depressive symptoms.

Results suggest that an easily accessible self-esteem treatment is feasible and effective for patients with a psychotic disorder. This is of great importance since approximately only one-third of patients receive psychological treatment [24]. Psychological treatment for psychosis is most often provided in an individual setting by a highly educated and specialized clinical psychologist [16]. This might be warranted for some forms of psychological treatment, but seems to be unnecessary for treatments addressing one specific symptom or characteristic, such as low self-esteem. Moreover, group interventions have important advantages: normalization of feelings, socialization and social support of people who are often isolated and lonely [25], and improvement of self-worth by helping others in the group [26]. Furthermore, psychotic disorders are associated with substantial economic costs for society and health care systems [27], which emphasizes the urgent need for cost-effective interventions. The current intervention is likely to reduce treatment costs.

Aside from the therapeutic setting, the treatment protocol by De Neef (2010) has not been investigated previously in patients with psychosis. The treatment protocol has been examined previously in an individual setting in a sample of patients with various psychiatric diagnoses including mood disorders, anxiety disorders, substance-related disorders, and personality disorders [28]. Results showed significant improvements in self-esteem as measured with the RSES. In the current study too, positive effects on the SERS as well as the RSES were found, while the RSES is viewed as a rather robust instrument that is not so sensitive to change [21]. Compared to previous investigated treatments targeting self-esteem in psychosis [6, 10, 12, 29], the current treatment protocol emphasizes behavioral elements. The current study indicates that such a structured program including concrete behavioral elements and practical exercises that are easily integrated into patients’ daily lives seems to be suitable for patients with psychosis.

While the present study results are promising, there are a number of limitations that need to be acknowledged. First, given the relatively small sample size, this pilot study provides only preliminary evidence. Furthermore, the study lacked a control group, so it cannot be ruled out that patients’ self-esteem and depression symptoms would have improved without the therapy. Also, it cannot be ruled out that medication use might have an effect on the results as well. Finally, the study design did not include follow-up assessments, so it is unclear whether the positive results were maintained. Future studies including a larger sample size, a control group, and follow-up assessments are warranted to allow for a more definite conclusion about the long-term benefits of the investigated group therapy.

## Data Availability

The datasets used and/or analysed during the current study are available from the corresponding author on reasonable request.

## Abbreviations

BDI-II: Beck Depression Inventory-II
CBT: Cognitive-behavioral therapy
COMET: Competitive memory training
DSM: Diagnostic and Statistical Manual of Mental Disorders
RSES: Rosenberg Self-Esteem Scale
SD: Standard deviation
SERS-SF: Self-Esteem Rating Scale - Short Form
SPSS: Statistical Package for the Social Sciences
UCP: University Center of Psychiatry
UMCG: University Medical Center Groningen
